# A Linguistic Survey of Cannabis Product Packaging Targeting University Students

**DOI:** 10.1192/j.eurpsy.2026.10876

**Published:** 2026-07-31

**Authors:** G. Fulkerson, B. J. Holstege, S. Nixon, R. Farah, C. P. Holstege

**Affiliations:** 1 Emergency Medicine, University of Virginia, Charlottesville; 2 Psychiatry, Old Dominion University, Norfolk, United States

## Abstract

**Introduction:**

Product packaging reveals intended impressions of the product and consumer characteristics. Prior researchers have evaluated cannabis products’ packaging appeal to children, but little has been studied about the university student population, many of whom cannot legally consume cannabis.

**Objectives:**

This study aims to more clearly delineate the type of cannabis product packaging geographically adjacent to a university and what it suggests about retailers’ target demographic.

**Methods:**

This convenience sample product survey considers cannabis products (n=55) purchased within a mile of a university from the time of decriminalization to the present. Product packages were evaluated manually; name and content labeling legal violations, warnings present, dosing information, and marketing language around selected topics - “natural”, “traditional”, “social”, “cool”, “risk to consumer”, and “legality of product” - were recorded.

**Results:**

Product types found were edibles (n=46), vapes (n=4), pre-rolls (n=3), oil (n=1), and incense (n=1). All but four of the edibles were sweet foods or candies. Gummies (n=16) and fruity candies (n=11) made up 59% of all edibles, demonstrating significant “sweet”, “candy”, and “fruity” preferences. These flavor preferences are all associated with youth. Forty-seven (86%) products have “youth appeal” features, most commonly “lookalike product” (n=28, 51%) and “taste descriptors” (n=28). Cartoons also featured prominently (n=19, 35%).

Thirty-eight products violated at least one of the cannabis name restrictions in state-level administrative code, the most common being “recreation” (n=38). The most prominently missing information is “use instructions” (71% missing). Twenty-two (40%) products omit cannabinoid content laboratory results, ten (18%) omit serving size, and thirteen (24%) omit warning labels. Thirty-six (65%) of products contained a “legal consumption age” warning. One product contained a “research purposes only” warning.

Sixteen products (29%) did not reference any of the six selected marketing topics. Six products (11%, 38% of products with no identified marketing language) came in unmarked clear packaging. “Legal” language surrounding the product’s delta-9 content or the 2018 Farm Bill was the most common product packaging language (n=35, 64%). “Natural” language surrounding sustainability, “vegan”, “organic”, or “all-natural” was the second-most common tactic (n=13, 24%).

**Conclusions:**

Cannabis product sellers located near a university target inexperienced consumers. Most product labels advertise the legality of their delta-9-tetrahydrocannabinol content. Minimal consumer benefits were specified on packaging. Youth appeal factors like fruity taste descriptors, edible gummy and candy formulations, and cartoon designs abound. Sporadically-printed use instructions, serving size listing, and warning labeling increase these inexperienced consumers’ risk of adverse health events.

**Disclosure of Interest:**

None Declared

